# Livogrit mitigates ANIT-induced cholestasis-like symptoms in an *in vivo* model by curbing hepatic inflammation and regulating BAX, TGF-β, MMP-9 and α-SMA gene expression

**DOI:** 10.1016/j.heliyon.2025.e41855

**Published:** 2025-01-11

**Authors:** Acharya Balkrishna, Ritu Paliwal, Surjeet Singh, Rani Singh, Vivek Gohel, Rishabh Dev, Kunal Bhattacharya, Sandeep Sinha, Anurag Varshney

**Affiliations:** a Drug Discovery and Development Division, Patanjali Research Foundation, Haridwar, 249 405, Uttarakhand, India; b Department of Allied and Applied Sciences, University of Patanjali, Patanjali Yog Peeth, Haridwar, 249 405, Uttarakhand, India; c Patanjali Yog Peeth (UK) Trust, 40 Lambhill Street, Kinning Park, Glasgow, G41 1AU, UK; d Special Centre for Systems Medicine, Jawaharlal Nehru University, New Delhi, 110 067, India

**Keywords:** Livogrit, Alpha-naphtylisothiocyanate, Cholestasis, Fibrosis, Inflammation, Ayurveda

## Abstract

Bile duct constriction disrupts bile acid flow causing cholestasis, hepatic necrosis, fibrosis, and cirrhosis. The study investigated hepatoprotective effectiveness of Ayurvedic prescription herbal medicine “Livogrit” commercially available in India, against α-naphtylisothiocyanate (ANIT)-induced cholestasis-like symptoms in male Sprague-Dawley rats. Livogrits's phytochemical profiling showed the presence of Gallic acid, Methyl Gallate, Catechin, Corilagin, Ellagic acid, Rutin, and Cinnamic acid. Sprague-Dawley rats were pre-treated with Livogrit (20–600 mg/kg/day) and reference drug Ursodeoxycholic acid (100 mg/kg/day) for 15 and 5 days, respectively, before single-dose ANIT (100 mg/kg) stimulation. Livogrit treatment protect the rats against ANIT-induced increase in blood serum markers (total bile acids, ALT, AST, GGT, ALP, total cholesterol, and total bilirubin) and reduced manifestation of liver necrosis, inflammation, and periportal fibrosis. At molecular level, Livogrit inhibited up-regulation of BAX, TGF-β, α-SMA, and MMP-9 mRNA expressions, associated with the liver damages. Taken together, this study supported Livogrit's potential as hepatoprotective medicine against cholestasis-like-etiologies.

## Graphical abstract

**Figure:**
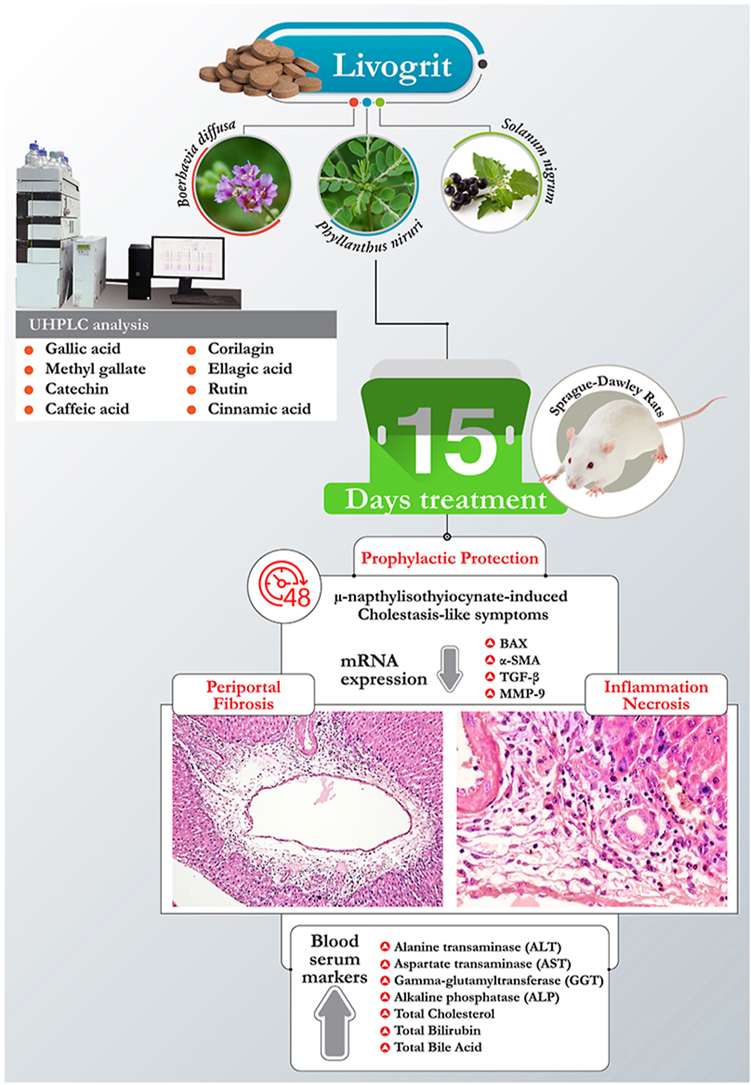

## 1 Introduction

Bile acids (BA) functions as an emulsifier in the duodenal region of the gastrointestinal tract, promoting the absorption of lipids, fats, and nutrients [1]. BA is primarily synthesized by the hepatocytes, and stored in gall bladder. This is then biologically converted into secondary BAs by intestinal microbiota [2]. The flow of BA from liver to intestine can be reduced or stalled due to various intrinsic and extrinsic factors such as, biliary atresia, drug-induced side effects, gallstone disease, pregnancy, sclerosing, and progressive familiar genetic deformities, resulting in cholestasis disease. Induction of cholestasis can lead to the accumulation of potentially toxic cholephiles in the liver and blood [3].

Under normal conditions, cholestasis does not produce any symptoms in patients, and only detected clinically through blood serum analysis for high levels of aspartate transaminase, alanine transaminase, gamma glutamyl transpeptidase, and alkaline phosphatase [4,5]. Other symptoms like pruritus, tiredness, and hyperbilirubinemia may appear during the later stages of disease progression. Prolonged existence of cholestasis may eventually lead to the development of cholangitis, progressive hepatic fibrosis, cirrhosis, and other functional failures, often necessitating liver transplantation [6]. Hepatic cholangitis is an inflammatory condition characterized by increased inflow of immune cells, and fibro-obliteration, resulting in fibrosis surrounding the bile duct [7]. Exposure to xenobiotic such as, alpha (α)-naphtylisothiocyanate (ANIT) can induce hepatic necrosis, chronic cholangitis, bile duct hyperplasia, and periportal fibrosis in rodents, mimicking cholestasis and cholangitis [[8], [9], [10]].

Livogrit is an Ayurvedic prescription medicine, commercially available throughout India, and formulated from root extract of *Boerhaavia diffusa* L., and whole plant extracts of *Phyllanthus niruri* L., and *Solanum nigrum* L mixed in the ratio of 2:1:1. The polyherbal medicine contains flavonoids, rotenoids, quercetin, kaempferol, alkaloids, anthocyanins, lignans, tannins, and steroidal glycosides obtained from its plant components [11]. Previous research have revealed substantial evidence for Livogrit's hepatoprotective action against toxic chemicals, and induced non-alcoholic and alcoholic fatty liver diseases [[11], [12], [13]].

**Fig. 1:**
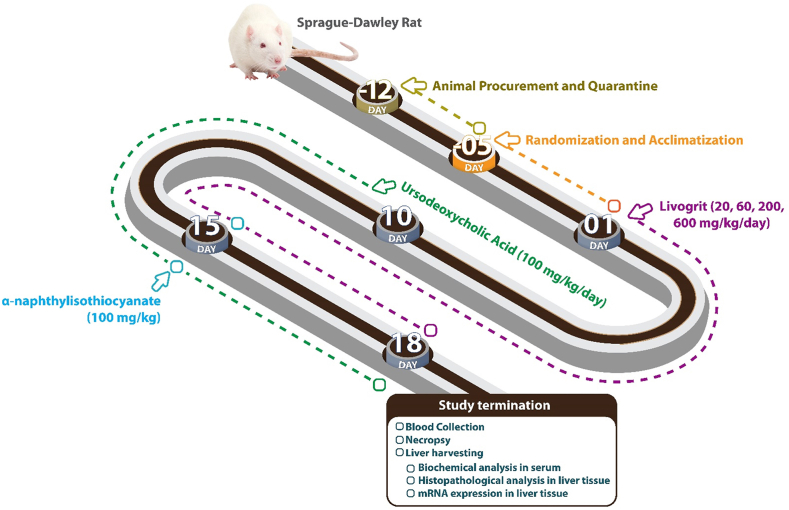
**Schematic of study design:** Male Sprague-Dawley rats were orally pre-treated with Livogrit 20–600 mg/kg/day for 15 days, and Ursodeoxycholic acid (100 mg/kg/day) for 5 days. Cholestasis-like symptoms were stimulated in the rats through a single-dose oral administration of 100 mg/kg of alpha-naphtylisothiocyanate (ANIT). After 48 h, the animals were sacrificed and biochemical, histopathological and molecular level biomarkers were analyzed in blood serum and harvested hepatic tissue samples.

In the present study, we have investigated the pharmacological effects of Livogrit on ANIT-induced cholestasis-like symptoms in male Sprague-Dawley rats. Animals were pre-treated with Livogrit, and the reference drug Ursodeoxycholic acid (UDCA) for 15 and 5 days, respectively. Later, rats were stimulated with a single dosage of ANIT, followed by continual Livogrit and UDCA treatments (Fig. 1). At the end of treatment regime, hepatic damage biomarkers were biochemically analyzed in blood serum of sacrificed rats. Hepatic tissue were also examined for pathological changes including the induction of necrosis, inflammation, and periportal fibrosis. The mRNA-level molecular markers for necrosis and fibrosis were also examined in the study groups. Finally, the observed hepatoprotective profile of Livogrit was linked with its rich phytochemical content that was evaluated using Ultra-High-Performance-Liquid-Chromatography (UHPLC) analytical technique.

## 2 Materials and methods

### 2.1 Reagents

Livogrit (Batch No. LGT220003) was sourced from Divya Pharmacy (Haridwar, Uttarakhand, India). Methyl cellulose was purchased from LobaChemie (Mumbai, Maharashtra, India). Alpha (α)-naphtylisothiocyanate (ANIT) and HPLC-grade Methyl Gallate (Potency, 97.3 %) were purchased from Tokyo Chemical Industry (India) Pvt. Ltd. (Hyderabad, Telangana, India). HPLC-grade acetonitrile was procured from Finar (Ahmedabad, Gujarat, India), methanol, diethylamine and orthophosphoric acid AR grade from Rankem (Pune, Maharashtra, India). Gallic acid standard (potency, 97.9 %), Ellagic acid (potency, 99.6 %) and Rutin (Potency, 94.2 %) were obtained from Sigma Aldrich (St. Louis, MO, USA). Corilagin (Potency, 98.0 %) was procured from Cayman Chemical Company (Ann Arbor, MI, USA), Cinnamic acid (Potency, 99.7 %) was purchased from SRL Pvt. Ltd (Mumbai, Maharashtra, India). Deionized water was obtained from a Milli Q system (Millipore, Billerica, MA, USA). RNAprotect tissue reagent and RNeasy Lysis Buffer were obtained from Qiagen (Hilden, Germany). Verso cDNA synthesis kit was purchased from ThermoFischer Scientific Inc. (Waltham, MA, USA). Primers for RT-PCR were purchased from Barcode Biosciences (Bangaluru, Karnataka, India). Harris hematoxylin stain was purchased from Sigma Aldrich. Eosin B solution was obtained from Himedia (Kennett Square, PA, USA). Total bile acid colorimetric quantification kit was purchased from Elabscience® (Houston, Texas, USA. SYBR-Green universal master mix was purchased from ThermoFischer Scientific Inc.

### 2.2 Ultra-High-Performance-Liquid-Chromatography (UHPLC) analysis

Gallic acid, Methyl Gallate, Catechin, Corilagin, Ellagic acid, Rutin, and Cinnamic acid standards were individually diluted in methanol for making 1 mg/ml stock solutions. Working standard containing all the phytochemical standards was generated by mixing them together equally (50 μg/ml). Separately, Livogrit tablets obtained from different batches (LGT210007, LGT210008, LGT210009, LGT210015, LGT210032) were powdered individually, and dissolved in methanol: water (80:20) solution. All the mixtures were sonicated for 30 min and centrifuged at 5000 rpm for 5 min. Resultant supernatants were collected, and filtered through nylon 0.45 μm syringe filter. These filtrates were then individually analyzed on Prominence-XR (Shimadzu, Japan) UHPLC platform equipped with Quaternary pump (NexeraXR LC-20AD XR), PAD detector (SPD-M20 A), Auto-sampler (Nexera XR SIL-20 AC XR), Degassing unit (DGU-20A 5R) and Column oven (CTO-10 AS VP) (Shimadzu, Kyoto, Japan).

Separation was achieved using a Shimadzu shim pack (3 μm, 3 × 100 mm) C18 column subjected to binary gradient elution. A gradient solvent system of solvent A (0.1 % orthophosphoric acid, pH 2.5 adjusted using diethyl amine), and solvent B (acetonitrile) was used for analysis. Gradient programming of the solvent system was initiated at 98 % solvent A for 0–4 min, followed by 98 to 90 % solvent A for 4–8 min, 90 to 85 % solvent A for 8–15 min, 85 % solvent A for 15–20 min, 85 to 80 % solvent A for 20–25 min, 80 to 65 % solvent A for 25–35 min, 65 to 5 % solvent A for 35–45 min, 5–98 % solvent A for 45 to 46 and 98 % solvent A for 46–50 min, with a flow rate of 0.5 ml/min. 4 μl of standard and test solution were injected for detection, and column temperature was maintain at 35 °C. Acquisition wavelength was adjusted to 270 nm, for these analytical experiments.

### 2.3 Animals and husbandry practices

Specific Pathogen Free (SPF) male Sprague-Dawley rats, aged 6–7 weeks were purchased from Vivo Bio Tech Ltd. (Hyderabad, Telangana, India, a Taconic Biosciences (Germantown, NY, United States) licensed animal supplier. The animals were quarantined for a week before initiation of the study in an animal house registered (1964/PO/Rc/S/17/CCSEA) with the Committee for Control and Supervision of Experiments on Animals, Department of Animal Husbandry and Dairying, Ministry of Fisheries, Animal Husbandry and Dairying, Government of India. At the end of quarantine period, all the animals were weighed using a pan balance (Sartorius, Goettingen, Germany) and randomly assigned to seven study groups of six animals each, based on their body weight. The animal groups were then acclimatized to the experimental region for five days. Animals were fed *ad libitum* with pelleted Purina 5L79 Charles River standard laboratory animal diet (Purina Animal Nutrition, Arden Hills, MN, USA) procured from Hylasco Biotechnology Pvt. Ltd. (Hyderabad, Telangana, India) and had unhindered access to reverse osmosis-treated drinking water. Animal house temperature was maintained at 22 ± 3 °C, and humidity levels between 30 and 70 %. Animal house's photoperiod was controlled automatically, set to 12 h of light and dark cycles, with 10–15 air changes per hour during the study period. The study protocol (PRIAS/LAF/IAEC-128) was approved by the Institutional Animal Ethical Committee (IAEC) of Patanjali Research Institute, Haridwar, Uttarakhand, India in compliance with CCSEA, Government of India guidelines.

### 2.4 Study design, formulation preparation and dosing

Every day before oral administration, the Livogrit powder was freshly triturated, weighed, and suspended in 0.5 % methyl cellulose (MC) solution at the required concentrations. Animal assigned to different treatment groups were treated accordingly. For the first fifteen days, assigned to NC and DC animals were given 0.5 % MC, b.i.d. orally on a daily basis till the end: For the first fifteen days, animals assigned to NC and DC were given 0.5 % MC, b.i.d. orally on a daily basis till the end of study. The RC group animals were orally fed with UDCA (100 mg/kg/day) for five days prior to disease induction and until the end of the experimental period. The recommended daily human dose of Livogrit is 2000 mg per day. Hence, its dosage in rats was estimated using the variations in body surface areas between the animal model and humans. Consequently, the human equivalent Livogrit dose was calculated at 206.67 mg/kg body weight, rounding it off to 200 mg/kg/day [14]. The other dosages were chosen to capture a plausible dose-response relationship, ranging from 1/10^th^ of the rat equivalent of human therapeutic dose to three times on a half-log scale. Half-log doses of Livogrit (20, 60, 200, and 600 mg/kg/day) were administered orally to the designated animal groups twice daily for fifteen days before disease induction and continued till the end of study. Dose volume was maintained at 5 ml/kg of body weight for all the animal groups, throughout the study duration. Except for NC, all the animal groups were given a single dose of ANIT (100 mg/kg), dissolved in Olive oil on designated day (Fig. 1). Earlier, ANIT has been used as a model stimulant in several earlier studies for the induction of cholestasis-like symptoms in Sprague-Dawley rats [15,16].

### 2.5 Blood sample collection

Animals were humanely sacrificed at the end of treatment regime through an intraperitoneal administration of thiopentone (150 mg/kg of body weight). Blood was extracted from the retro-orbital plexus in sterile collecting vials and allowed to form a clot standing at RT for 45 min. Serum was isolated by centrifuging clotted blood at 2000×*g* for 10 min at 4 °C using a refrigerated centrifuge (Sorvall ST 8R; Thermo Fisher Scientific Inc.), and stored at −80 °C till further use.

### 2.6 Hepatic tissue isolation and processing

Hepatic tissue was harvested surgically from the sacrificed animals. One lobe of liver was fixed in 10 % neutral buffered formalin for histopathological evaluation and the second lobe was stored in RNAprotect tissue reagent and flash frozen in liquid nitrogen before storing at −80 °C. For histopathological investigation, formalin-treated tissue was dehydrated using different concentrations of ethanol and xylene in an automated TP-1020 tissue processor (Leica Biosystems, Nussloch, Germany) and embedded in paraffin wax using a Histocore Arcadia H-C embedding station (Leica Biosystems). Tissue sections of 3–5 μm thickness were prepared using a RM 2245 microtome (Leica Biosystems). Tissue sections were stained with Harris hematoxylin stain/Eosin B stains for inflammation and necrosis analysis, and with Masson's Trichrome stain for collagen staining. All the stained tissues were microscopically examined using a brightfield Olympus BX43 microscope (Olympus corporation, Tokyo, Japan) equipped with Mantra imaging platform (PerkinElmer, MA, USA). Blinded scoring was performed following Li et al. with slight modification (0 = normal tissue; 1 = Minimal (<10 % of hepatocytes); 2 = Mild (10–50 % of hepatocytes); 3 = Moderate (50–75 % of hepatocytes); 4 = Severe (>75 % of hepatocytes), by a trained histopathologist for determining occurrence and severity of hepatic necrosis, inflammation, and periportal fibrosis [17].

### 2.7 Serum biochemical analysis

Frozen serum samples were thawed to room temperature and analyzed for levels of aspartate transaminase (AST), alanine transaminase (ALT), gamma-glutamyl transferase (GGT), alkaline phosphatase (ALP), total cholesterol (CHOL) and total bilirubin (TBIL) using EM-200 biochemistry analyzer (Erba, Mannheim, Germany), following manufacturer's instructions. The total serum bile acid level was measured at 405 nm using an Infinite 200Pro plate reader (Tecan, Switzerland) and a Total Bile Acid Colorimetric Assay Kit (Elabscience®) according to the manufacturer's instructions.

### 2.8 Real-time PCR analysis

Second lobe of liver tissue samples stored in RNAprotect tissue reagent were thawed and processed for isolation of total RNA using RNeasy Lysis Buffer at 4 °C. Complementary DNA (cDNA) was synthesized using a one-step Verso cDNA synthesis kit. For RT-PCR, primer used were:

BAX: F- 5′ CAGACGGCAACTTCAACTGG 3’

R- 5′ GGTCCCGAAGTAGGAAAGGAG 3’

α-SMA: F- 5′ ATAGAACACGGCATCATCACC 3’

R- 5′ GGTCTCAAACATAATCTGGGTCA 3’

TGF β-1: F- 5′ CTGAACCAAGGAGACGGAAT 3’

R- 5′ GGTTCATGTCATGGATGGTG 3’

MMP-9: F- 5′ AAACCTCCAACCTCACGGAC 3’

R- 5′ TGGCCTTTAGTGTCTCGCTG 3’

GAPDH: F- 5′ ATGTTTGTGATGGGTGTGAA 3’

R- 5′ ATGCCAAAGTTGTCATGGAT 3’

Primer sets were combined individually with SYBR-Green universal master mix before the RT-PCR analysis using a Biometra Professional RT-PCR equipment (Analytik-Jena AG, Jena, Germany). RT-PCR analysis was done using the following parameters: denaturation at 95 °C for 10 min, annealing at 60 °C for 1 min, and extension at 75 °C for 15 s during a 40-cycles run. GAPDH was used as a house-keeping gene. Results were analyzed as relative mRNA expression fold changes compared to the house-keeping gene [18].

### 2.9 Statistical analysis

All the data for the studied parameters were compiled from each of the study groups and expressed as mean ± standard error of mean (SEM). Statistical analysis was performed using GraphPad Prism version 7.04 software (GraphPad Software, San Diego, CA, USA). One-way analysis of variance (ANOVA) followed by Dunnett's multiple comparison post-hoc test was employed to calculate the statistical differences between the study groups. p-value variation of <0.05 was considered to be statistically significant.

## 3 Results

### 3.1 Ultra-High-Performance-Liquid-Chromatography (UHPLC) analysis

**Fig. 2:**
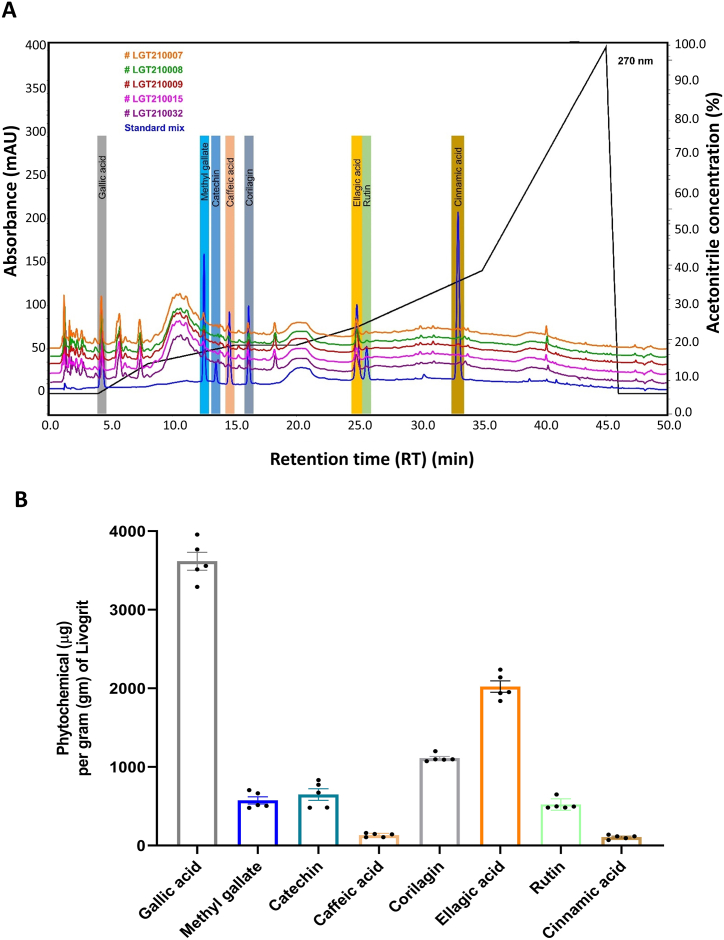
**Ultra-High Performance Liquid Chromatography (UHPLC) analysis:** Five batches of Livogrit were analyzed individually using UHPLC technique. A) Chromatogram showing the identification of Gallic acid (RT 4.31 min), Methyl Gallate (RT 12.56 min), Catechin (RT 13.50 min), Corilagin (RT 16.16 min), Ellagic acid (RT 24.93 min), Rutin (RT 25.67 min), and Cinnamic acid (RT 33.13 min) contents. B) Graphical representation for the quantity (mean ± standard deviation) of individual phytochemicals represented as in phytochemical (microgram (μg)) per gram (gm) of Livogrit.

UHPLC analysis of the selected Livogrit batches showed the presence of Gallic acid (3616.60 ± 254.03 μg per gram of Livogrit (μg/gm) at RT 4.31 min; Methyl Gallate (573.59 ± 103.02 μg/gm) at RT 12.56 min; Catechin (648.48 ± 162.87 μg/gm) at RT 13.50 min; Caffeic acid (131.42 ± 23.92 μg/gm) at RT 14.59 min; Corilagin (1111.96 ± 49.35 μg/gm) at RT 16.16 min; Ellagic acid (2021.41 ± 162.00 μg/gm) at RT 24.93 min; Rutin (521.48 ± 71.68 μg/gm) at RT 25.67 min; and Cinnamic acid (106.85 ± 26.12 μg/gm) at RT 33.13 min (Fig. 2A and B). Phytochemicals such as, Gallic acid, Methyl Gallate, Catechin, Caffeic acid, Corilagin, Ellagic acid, Rutin, and Cinnamic acid are known for their anti-inflammatory behavior [[19], [20], [21], [22], [23], [24], [25], [26]].

### 3.2 Biochemical analysis

**Fig. 3:**
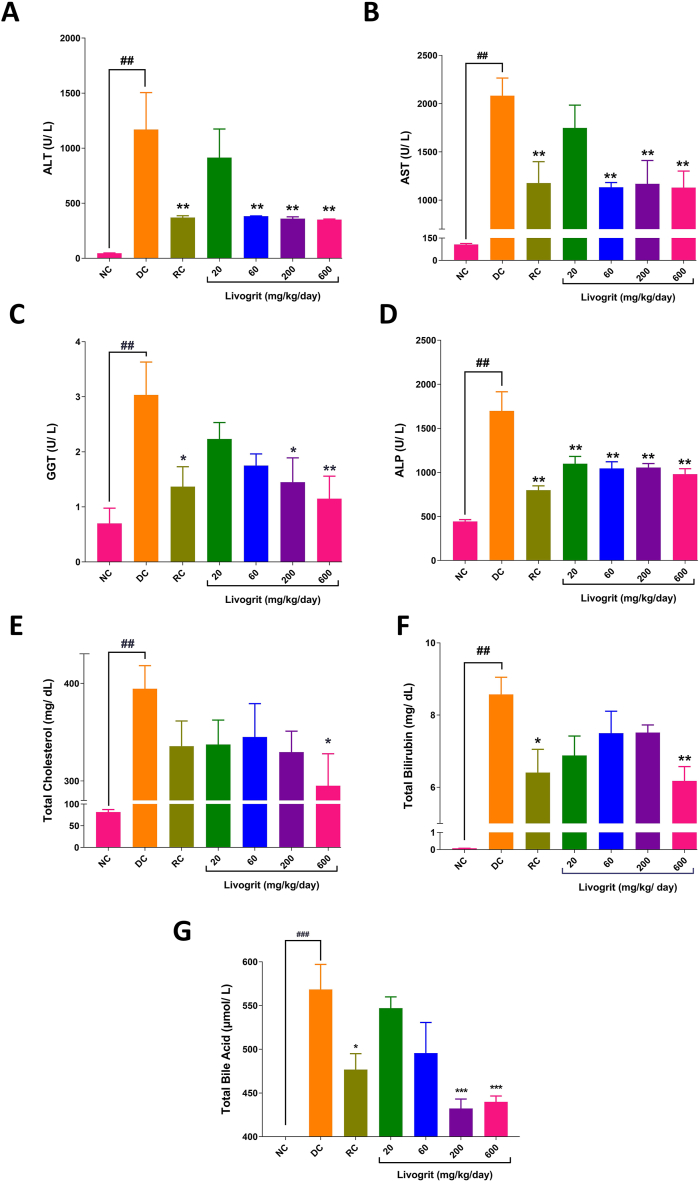
**Biochemical analysis:** Blood serum collected from normal control (NC); disease control (DC) animals stimulated with ANIT (100 mg/kg); Livogrit (20, 60, 200, and 600 mg/kg/day), and Ursodeoxycholic acid (RC) (100 mg/kg/day) pre-treated animals stimulated with ANIT (100 mg/kg) were analyzed for (A) alanine transaminase (ALT), (B) aspartate transaminase (AST), (C) glutamyltranspeptidase (GGT), (D) alkaline phosphatase (ALP), (E) total cholesterol, (F) total bilirubin, and (G) total bile acid. Number of animals per study group was six. Results have been expressed as mean ± standard error of mean (SEM). Statistical analysis was performed using one-way ANOVA followed by Dunnett's posthoc test. ## p-value <0.01 (NC *versus* DC); ∗ p-value <0.05 (RC/Livogrit treatment groups *versus* DC); ∗∗ p-value <0.01 (RC/Livogrit treatment groups *versus* DC); ∗∗∗ p-value <0.001 (Livogrit treatment groups *versus* DC).

In this study, the normal control (NC) Sprague-Dawley rats showed a baseline levels of ALT, AST, GGT, ALP, total cholesterol, total bilirubin and total bile acid in the blood serum (Fig. 3A–G). These were within the laboratory background values reported in-house and by Delwatta et al. for Sprague-Dawley rats [27].Stimulation of the rats with α-naphtylisothiocyanate (ANIT) causes cholestasis-like inflammation, fibrosis, and biochemical alterations in livers by directly interacting with hepatocytes and bile duct epithelial cells [9,28]. ANIT induction in disease control (DC) animals significantly (p-value <0.01) increased blood serum levels of ALT, AST, GGT, ALP, total cholesterol, total bilirubin and total bile acid (Fig. 3A–G). Treatment of rats with varying concentrations of Livogrit provided significant (p-value <0.05) protection against ANIT-stimulated hepato-stress by lowering ALT levels (60, 200, and 600 mg/kg/day), AST (60, 200, and 600 mg/kg/day), GGT (200, and 600 mg/kg/day), ALP (20, 60, 200, and 600 mg/kg/day), total cholesterol (600 mg/kg/day), total bilirubin (600 mg/kg/day), and total bile acid (200, and 600 mg/kg/day), compared to DC animals (Fig. 3A–G). Rats treated with UDCA (RC) also showed significant (p-value <0.05) protection against ANIT-induced alterations in blood serum biochemical parameter (Fig. 3A–G).

### 3.3 Histopathological analysis

**Fig. 4:**
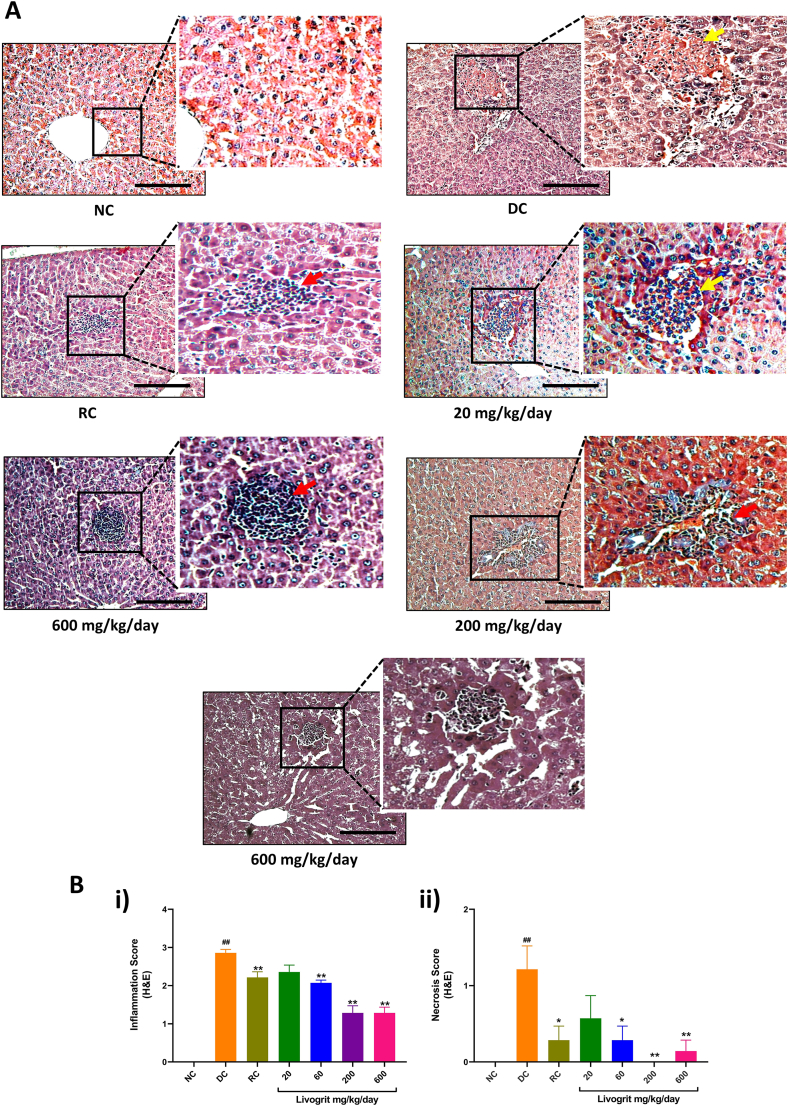
**Histopathological analysis for tissue inflammation and necrosis:** (A) Harris hematoxylin, and eosin B stained liver tissue sections were analyzed for the presence of inflammatory cells (red arrow), and necrotic patches (yellow arrows) in normal control (NC); disease control (DC) animals stimulated with ANIT (100 mg/kg); Livogrit (20, 60, 200, and 600 mg/kg/day), and Ursodeoxycholic acid (RC) (100 mg/kg/day) pre-treated animals stimulated with ANIT (100 mg/kg). Images were acquired at 100 × magnification. Image scale bars represent 100 μm. (B) Blinded severity scoring was done for determining i) inflammation, and ii) hepatic necrosis scores in NC, DC, Livogrit (20, 60, 200, and 600 mg/kg/day), and Ursodeoxycholic acid (RC) (100 mg/kg/day) pre-treated animals stimulated with ANIT (100 mg/kg). Number of animals per study group was six. Results have been expressed as mean ± standard error of mean (SEM). Statistical analysis was performed using one-way ANOVA followed by Dunnett's posthoc test. ## p-value <0.01 (NC *versus* DC); ∗ p-value <0.05 (RC/Livogrit treatment groups *versus* DC); ∗∗ p-value <0.01 (RC/Livogrit treatment groups *versus* DC).

Single dose administration of ANIT in DC Sprague-Dawley rats induced significant (p-value <0.01) increase in inflammation score, necrosis score, and periportal fibrosis score within 48 h, compared to NC animals (Fig. 4A and B(i), 4B(ii), 5A and 5B). Livogrit treatment of the rats at varying concentrations significantly (p-value <0.01) protected them against the ANIT-stimulated induction of hepatic injuries, observed through improved inflammation score (60, 200 and 600 mg/kg/day), necrosis score (60, 200 and 600 mg/kg/day), and periportal fibrosis score (200 and 600 mg/kg/day) (Fig. 4B(i), 4B(ii), 5A and 5B). RC animals treated with UDCA also showed a statistically significant (p-value <0.01) protection against ANIT-induced inflammation, necrosis and periportal fibrosis (Fig. 4B(i), 4B(ii), 5A and 5B).

**Fig. 5:**
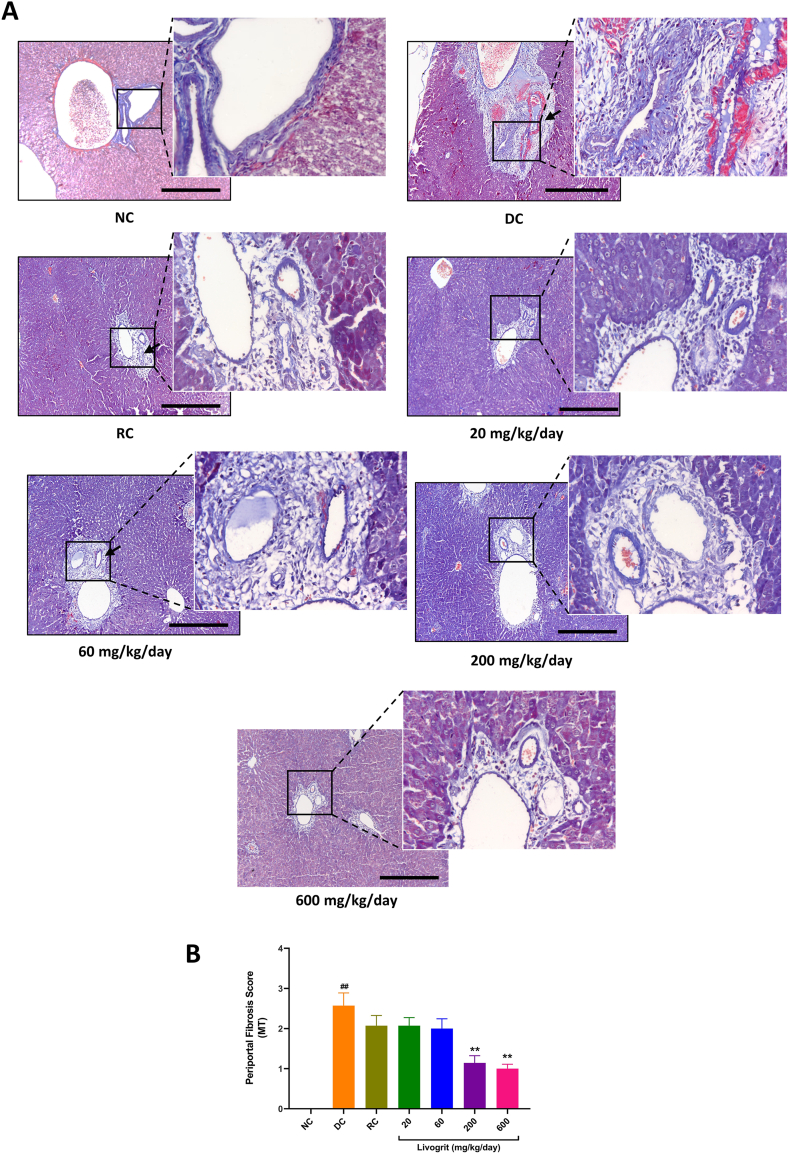
**Histopathological analysis for tissue fibrosis:** (A) Masson's Trichrome stained liver tissues of experimental rats were analyzed for periportal fibrosis in normal control (NC); disease control (DC) animals stimulated with ANIT (100 mg/kg); Livogrit (20, 60, 200, and 600 mg/kg/day), and Ursodeoxycholic acid (RC) (100 mg/kg/day) pre-treated animals stimulated with ANIT (100 mg/kg). Black arrow indicates the location for periportal fibrosis. Images were acquired at 100 × magnification. Image scale bars represent 100 μm. (B) Blinded severity scoring was done for determining extent of hepatic periportal fibrosis score in NC, DC, Livogrit (20, 60, 200, and 600 mg/kg/day), and Ursodeoxycholic acid (RC) (100 mg/kg/day) pre-treated animals stimulated with ANIT (100 mg/kg). Number of animals per study group was six. Results have been expressed as mean ± standard error of mean (SEM). Statistical analysis was performed using one-way ANOVA followed by Dunnett's posthoc test. ## p-value <0.01 (NC *versus* DC); ∗∗ p-value <0.01 (Livogrit treatment groups *versus* DC).

### 3.4 Gene expression changes

**Fig. 6:**
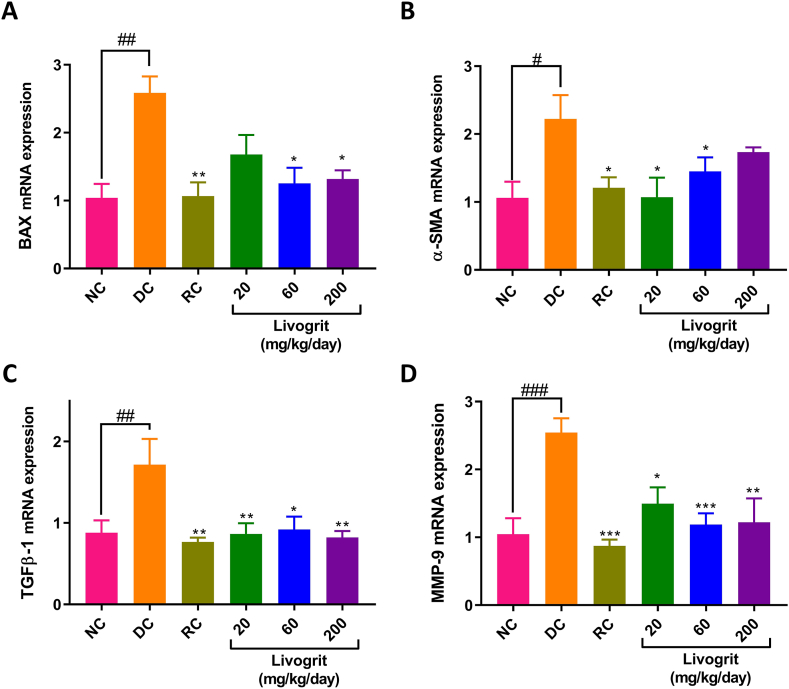
**Gene expression analysis:** RT-PCR analysis was performed in the liver tissues of normal control (NC); disease control (DC) animals stimulated with ANIT (100 mg/kg); Livogrit (20, 60, 200, and 600 mg/kg/day), and Ursodeoxycholic acid (RC) (100 mg/kg/day) pre-treated animals stimulated with ANIT (100 mg/kg) for determining mRNA expression levels of (A) BAX, (B) α-SMA, (C) TGF-β1, and (D) MMP-9. Number of animals per study group was six. Results have been expressed as mean ± standard error of mean (SEM). Statistical analysis was performed using one-way ANOVA followed by Dunnett's posthoc test. # p-value <0.05 (NC *versus* DC); ## p-value <0.01 (NC *versus* DC); ### p-value <0.001 (NC *versus* DC); ∗ p-value <0.05 (RC/Livogrit treatment groups *versus* DC); ∗∗ p-value <0.01 (RC/Livogrit treatment groups *versus* DC); ∗∗∗ p-value <0.001 (RC/Livogrit treatment groups *versus* DC).

In DC rats, ANIT-stimulation significantly (p-value <0.05) increased mRNA expressions of BAX, α-SMA, TGF-β, and MMP-9, compared to NC animals (Fig. 6A–D). Livogrit treatment at the concentrations of 20, 60, 200 and 600 mg/kg/day in the ANIT-treated rats, reduced mRNA expression levels of BAX, α-SMA, TGF-β, and MMP-9, compared to DC animals (Fig. 6A–D). UDCA treatment (RC) also significantly (p-value <0.01) protected the rats against ANIT-induced increase in mRNA expression levels of BAX, α-SMA, TGF-β, and MMP-9 (Fig. 6A–D).

Overall, the study found that Livogrit and UDCA treatments protected the Sprague-Dawley rats from ANIT-induced liver injuries. Livogrit treatment reduced ANIT-stimulated infiltration of hepatic tissue by inflammatory cells decreasing inflammation, development of fibrosis, and necrosis.

## 4 Discussion

Bile acids (BA) play the pivotal role in lipid and nutrient absorption in the duodenum. Cholestasis induces intrahepatic (within the liver) or extrahepatic (outside the liver) impairment of bile flow, resulting in the accumulation of bile acids and toxic cholephiles within the liver [29]. Intrahepatic cholestasis may lead to dysfunction of hepatocytes or the bile canaliculi, while extrahepatic cholestasis typically results from obstruction of the bile ducts outside the liver. This condition poses a significant challenge to normal hepatic function, and can lead to a spectrum of mild to severe metabolic complications.

In the present study, α-naphthylisothiocyanate (ANIT) functioned as a stimulant for inducing cholestasis-like symptoms in the rat liver. Inside the liver, ANIT undergoes metabolism and temporarily conjugates with glutathione, thereby losing its toxicity. Free-form of ANIT exerts toxicity on the bile duct epithelial cells and hepatocytes causing cellular damages, and necrosis [30]. ANIT only causes intrahepatic cholestasis-like damages and inflammation [30]. In our study, a single dosage of ANIT at 100 mg/kg elicited an increase in the blood serum ALT, AST, GGT, ALP, total cholesterol, total bilirubin and total bile acid levels in male Sprague-Dawley rats. These findings are consistent with previous investigations showing ANIT-induced cholestasis through alteration in the blood serum biochemical markers [31,32]. In our investigation, the increase in biochemical markers was associated with hepatic tissue damage produced by ANIT-stimulation, such as necrosis, inflammatory cell influx, and periportal fibrosis.

Livogrit is a herbal Ayurvedic prescription medicine formulated with extracts obtained from root extract of *Boerhaavia diffusa* L., and whole plant extracts of *Phyllanthus niruri* L., and *Solanum nigrum* L mixed in the ratio of 2:1:1. Previously, Livogrit was demonstrated to have the hepatoprotective effect against thioacetamide-induced hepatocellular damage in Zebrafish model, and in human hepatocyte spheroids against chemically-induced non-alcoholic, and fatty acid hepatosteatosis models [12,13,33]. In the present study, Livogrit protected the Sprague-Dawley rats from ANIT-induced hepatic injuries, as shown by substantial reduction in blood serum levels of ALT, AST, GGT, and total bile acid majorly between the therapeutic dosages of 60–600 mg/kg/day including the human equivalent dose. Total bilirubin level, and total cholesterol serum levels stimulated by ANIT were reduced at all the tested dosages of Livogrit with maximum reduction observed at highest concentration of 600 mg/kg/day. The results correlated well with the histopathological observations made in the hepatic tissue of the ANIT-treated DC animals showing induction of necrotic patches and inflammation.

At the molecular level, ANIT has been shown to increase the mRNA expression levels of BAX, α-SMA, TGF-β, and MMP-9. Bajt et al. have shown that in response to hepatotoxic chemical exposure and induction of free radicals, the BAX protein is translocated from cytosol to mitochondria in hepatocyte [34]. This leads to DNA fragmentation, and cellular necrosis indicated through an increase in the presence of blood serum ALT [34]. Necrotic hepatocytes release damage-associated molecular patterns due to plasma membrane damage, that stimulate the hepatic stellate and the Kuepfer cells to generate TGF-beta (TGF-β), and alpha-smooth muscle actin (α-SMA) leading to hepatocyte fibroblast activation, and initiation of hepatic fibrosis [35,36]. Matrix metalloproteases (MMP) are calcium-dependent zinc-containing endo-proteinases that play an important role in hepatic inflammation, fibrosis and rejuvenation [37]. MMP-9, also known as ‘Gelatinase-B’, is secreted by a variety of immune and fibroblast cell types, and can act differently depending on the stage and etiology of hepatic diseases. In the present study, Livogrit treatment at all the tested dosages inhibited the ANIT-stimulated increase in the mRNA expression levels of BAX, α-SMA, TGF-β, and MMP-9.

Previous study with *Boerhaavia diffusa* L. plant extract has shown its hepatoprotective properties against the chemotherapeutic agent Oxaliplatin through a reduction in blood serum ALT, AST, and GGT levels [38]. However, the study also observed that *Boerhaavia diffusa* L. plant extract treatment did not modulate the total bilirubin level [38]. *Phyllanthus niruri* L. is capable of reducing the serum levels of total cholesterol, ALT, and ALP along with total bilirubin [39,40].

Phytochemicals like Gallic acid, Methyl Gallate, Catechin, Caffeic acid, Corilagin, Ellagic acid, Rutin, and Cinnamic acid that were quantified in the Livogrit using Ultra-High Performance Liquid Chromatography technique, are known to provide hepatoprotection against various chemical insults [19,21,22,25,26,41,42]. Studies have shown Gallic acid and Ellagic acid to ameliorate hepatic inflammation, and protect against liver injuries and fibrosis [19,41,43]. Gallic acid and Corilagin has been found to inhibit the activation and proliferation of hepatic stellate cells leading to the inhibition of TGF-β, and α-SMA expression, along with leading to recovery of the antioxidant levels [43,44]. Rutin suppressed the expression of Toll like-receptors and P2X7 receptors in hepatic stellate cells of mice, disrupting their activation and initiation of fibrogenesis [45]. Therefore, these phytochemicals may be involved in the protective effect of Livogrit against inflammation, necrosis and fibrosis induced by ANIT-stimulation.

Innate immune cells play a major role in the hepatic injuries by presenting a complex set of defense mechanism involving inflammation, fibrosis and liver regeneration. Following chemical or pathological insults, resident macrophages and dendritic cells release cytokines in response to pathogen assisted molecular patterns and damage associated molecular patterns by releasing cytokines and chemokines. Neutrophils are the first phagocytes to arrive at the site of inflammation and become activated releasing cytotoxic antimicrobial molecules [46]. Inflammatory neutrophils produce MMP-9, as a signaling molecule that promote further recruitment at the site of injury. These recruited neutrophils mediate damage to the surround hepatic tissue through generation or reactive oxygen species causing oxidative stress in a mitogen-activated protein kinase-activating protein kinase 2-dependent manner and the production of MPO [47]. In the present study, ANIT induced an influx of inflammatory cells that was also represented by the increase in hepatic tissue MMP-9 mRNA expression level. Pre-treatment of the rats with Livogrit at concentrations between 60 and 600 mg/kg/day, including human equivalent dosages significantly reduced the influx of neutrophils and the enhanced expression level of MMP-9. The results indicated the anti-inflammatory activity of the Livogrit as has been earlier demonstrated by amelioration of lymphocytic influx and fibrosis in liver tissue following carbon tetrachloride treatment of Wistar rats [11].

Overall, Livogrit showed hepatoprotective benefits in Sprague-Dawley rats against ANIT-induced cholestasis-like hepatic inflammation at all the tested dosages, including human equivalent dosage of 200 mg/kg/day. Only exception was observed in the modulation of total cholesterol and total bilirubin markers that required a higher dose (600 mg/kg/day) of Livogrit. Hence, Livogrit's ability to mitigate biochemical, histopathological, and molecular markers associated with cholestasis suggests a multi-faceted protective mechanism.

The phytochemical makeup of Livogrit, as identified by UHPLC analysis, most certainly contributes to its medicinal benefits. Overall, Livogrit showed hepatoprotective benefits in Sprague-Dawley rats against ANIT-induced cholestasis-like hepatic inflammation at all the tested dosages, including human equivalent dosage of 200 mg/kg/day. Hence, Livogrit's ability to mitigate biochemical, histopathological, and molecular markers associated with cholestasis suggests a multi-faceted protective mechanism. The phytochemical makeup of Livogrit, as identified by UHPLC analysis, most certainly contributes to its medicinal benefits.

In conclusion, Livogrit emerges as a promising candidate for further exploration in the context of hepatic diseases, particularly cholestasis. Its hepatoprotective activity originated from the presence of phytochemicals, that controlled the inflammation, necrosis and fibrosis processes at the molecular levels. This disrupted the induction of cholestasis in the Sprague-Dawley rats treated with the ANIT and reduced their blood serum biomarkers for hepatic injury, and reduced total cholesterol and bile acid concentrations. Therefore, the present study presented Livogrit as a promising herbal Ayurvedic medicine, capable of providing hepatic protection against drug and chemical induced cholestasis. The study requires further investigation for understanding the underlying mode of actions and therapeutic potential of Livogrit in cholestasis patients.

## 5 Limitation of the study

Further research is required for determining the biological mechanisms and signaling pathways involved in Livogrit's hepatoprotective activity. Nonetheless, these findings highlight the potential of traditional medicines such as, Livogrit in as an alternative or complementary route for the treatment of cholestasis –like hepatic disorders.

## 6 Animal study ethical permission

The study protocol (PRIAS/LAF/IAEC-128) was approved by the Institutional Animal Ethical Committee (IAEC) of Patanjali Research Institute, Haridwar, Uttarakhand, India in compliance with CCSEA, Government of India guidelines.

## CRediT authorship contribution statement

**Acharya Balkrishna:** Writing – review & editing, Visualization, Supervision, Conceptualization. **Ritu Paliwal:** Investigation, Formal analysis, Data curation. **Surjeet Singh:** Formal analysis, Data curation. **Rani Singh:** Investigation, Formal analysis, Data curation. **Vivek Gohel:** Investigation, Formal analysis, Data curation. **Rishabh Dev:** Writing – review & editing, Methodology, Investigation, Formal analysis, Data curation. **Kunal Bhattacharya:** Writing – original draft, Visualization, Data curation. **Sandeep Sinha:** Methodology, Investigation, Formal analysis, Data curation. **Anurag Varshney:** Visualization, Supervision, Project administration, Conceptualization.

## Data availability statement

All raw data are available on request from corresponding author.

## Funding

This study has been funded internally by Patanjali Research Foundation Trust, Haridwar, India.

## Declaration of competing interest

The authors declare the following financial interests/personal relationships which may be considered as potential competing interests: Acharya Balkrishna reports a relationship with Divya Pharmacy, Haridwar, Uttarakhand that includes: board membership. Livogrit was sourced from Divya Pharmacy, Haridwar, Uttarakhand, India. Acharya Balkrishna is an honorary trustee in Divya Yog Mandir Trust, which governs Divya Pharmacy, Haridwar. In addition, he holds an honorary managerial position in Patanjali Ayurved Ltd., Haridwar, India, Divya Pharmacy and Patanjali Ayurved Ltd commercially manufacture and sell several Ayurvedic products. Other than supplying the test article, Divya Pharmacy was not involved in any aspect of the research stated in this study. Authors Ritu Paliwal, Surjeet Singh, Rani Singh, Vivek Gohel, Rishabh Dev, Kunal Bhattacharya, Sandeep Sinha and Anurag Varshney have been employed by Patanjali Research Foundation Trust (PRFT), Haridwar, Uttarakhand, India. PRFT is an independent not-for-profit research organization. The remaining authors declare no competing interests. If there are other authors, they declare that they have no known competing financial interests or personal relationships that could have appeared to influence the work reported in this paper.
